# Indole-3-carbinol: a plant hormone combatting cancer

**DOI:** 10.12688/f1000research.14127.1

**Published:** 2018-06-01

**Authors:** Ella Katz, Sophia Nisani, Daniel A. Chamovitz

**Affiliations:** 1 School of Plant Sciences and Food Security, Tel Aviv University, Tel Aviv, Israel; 2 Department of Plant Sciences, University of California , Davis , USA

**Keywords:** glucosinolates, indole-3-carbinol, cancer prevention, cruciferous vegetables

## Abstract

A diet rich in cruciferous vegetables such as cauliflower, broccoli, and cabbage has long been considered healthy, and various epidemiological studies suggest that the consumption of cruciferous vegetables contributes to a cancer-protecting diet. While these vegetables contain a vast array of phytochemicals, the mechanism by which these vegetables counteract cancer is still largely unresolved. Numerous *in situ* studies have implicated indole-3-carbinol, a breakdown product of the glucosinolate indole-3-ylmethylglucosinolate, as one of the phytochemicals with anti-cancer properties. Indole-3-carbinol influences a range of cellular processes, but the mechanisms by which it acts on cancer cells are slowly being revealed. Recent studies on the role of indole-3-carbinol in Arabidopsis opens the door for cross-kingdom comparisons that can help in understanding the roles of this important phytohormone in both plant biology and combatting cancer.

## Introduction

A diet rich in cruciferous vegetables such as cauliflower, broccoli, and cabbage has long been considered healthy. Even in ancient times, extracts from these vegetables were thought to have medicinal and curative properties, and both Pythagoras and Hippocrates understood the medicinal properties of mustard extracts ^1^. In the 20 ^th^ century, epidemiological studies pointing to the protective properties of cruciferous vegetables in a cancer-protecting diet started to accumulate ^2^. A meta-analysis of studies carried out over 18 years in Europe revealed an inverse association between weekly consumption of cruciferous vegetables and several common cancers, including colorectal, breast, kidney, and upper digestive tract cancers ^3^. While these vegetables contain a vast array of phytochemicals ^4^, the mechanism by which these vegetables counteract cancer is still largely unresolved.

The anticarcinogenic properties associated with crucifers are primarily attributed to the presence of glucosinolates, a family of secondary metabolites that are synthesized uniquely in this plant family and play a dominant role in plant defenses against insects ^5^. Glucosinolates are derived from glucose and amino acids and contain various modifications to their side chain. The exact glucosinolate profile varies among crucifer species, and more than 140 glucosinolates have been characterized, including approximately 40 in Arabidopsis ^6, 7^. Herbivory or other tissue damage initiates the hydrolysis of glucosinolates by an endogenous plant β-thioglucosidase termed “myrosinase”. Glucosinolates and myrosinase are stored in separate plant compartments and meet only following cell rupture. This separation is likely an adaptation to allow the targeted production of glucosinolate breakdown products, which are toxic to not only herbivores and pathogens but also the plants themselves. Further catalysis and spontaneous degradation results in the formation of nitriles, epithionitriles, oxazolidine-2-thiones, thiocyanates, and isothiocyanates ^8, 9^. These glucosinolate breakdown products cause the characteristic sharp taste of cruciferous vegetables and typically have a deterrent effect on generalist herbivores ^10– 12^.

**Figure 1.**
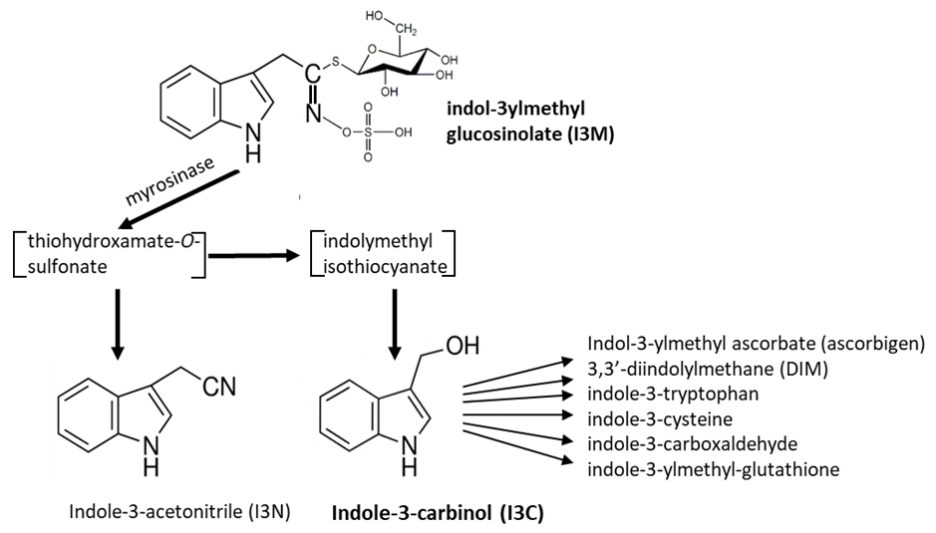
Myrosinase-catalyzed breakdown of indol-3-ylmethyl­glucosinolate (I3M-GS). Myrosinase-catalyzed breakdown of I3M-GS leads to the formation of unstable intermediates and then to indole-3-acetonitrile (I3N) and indole-3-carbionol (I3C). I3C reacts with itself and other plant metabolites to form a number of conjugates, some of which are shown.

Breakdown of indole-3-ylmethylglucosinolate (I3M-GS), one of the most widely distributed glucosinolates, leads to the formation of indole-3-acetonitrile (I3N) and indole-3-carbinol (I3C) ( Figure 1) ^13^. I3C, in turn, can react with itself and a variety of other plant metabolites to form conjugates, some of which are shown in Figure 1. Most of these I3C conjugates have as-yet-unknown functions in plant metabolism, though, interestingly, another function of glucosinolate breakdown products may be to signal further plant defense responses ^14^. Therefore, it is possible that I3M-GS breakdown also triggers other downstream responses in Arabidopsis and other crucifers.

From a dietary perspective, cooking vegetables affects their profile of glucosinolate breakdown ^15^. Boiling leads to inactivation of the myrosinase enzyme but can also lead to a non-enzymatic breakdown of I3M-GS to I3C and I3N ^16^. In addition, human gut microbes can lead to glucosinolate breakdown ^17^.

## Indole-3-carbinol and cancer

The glucosinolate breakdown products, rather than intact glucosinolates, primarily contribute to the anticarcinogenic effects of eating cabbage, broccoli, and related vegetables ^11, 12, 18^. I3C has long been studied regarding potential roles in cancer management ^19, 20^, and many studies showed that I3C suppresses the proliferation of various cancer cell lines, including breast, colon, prostate, and endometrial cancer cells (reviewed in 19, 21). One example of its anti-proliferative properties comes from a study conducted on non-tumorigenic and tumorigenic breast epithelial cells (MCF10A and MCF10CA1a, respectively), which showed that I3C induced apoptosis in the breast cancer cells but not in the non-tumorigenic breast epithelial cells ^22^. I3C and one of its reaction products, diindolylmethane (DIM), were implicated in the induction of phase 1 detoxification enzymes, which can result in the breakdown of other dietary carcinogens. Both *in situ* and *in vivo* studies point to a role for I3C as a chemoprotective agent in breast and prostate cancer ^23^.

The exact mechanisms by which I3C influences human cells are unclear, though direct interaction with a variety of signaling pathways has been proposed. The treatment of various cancer cells with I3C induces G1 cell cycle arrest ^24– 30^. Other studies pointed to a connection between treatment of I3C and stimulation of apoptosis in several tumor cells ^25, 31– 34^. I3C also induced autophagy in different cell lines. For example, the treatment of human colon cancer HT-29 cells with I3C and genistin induced autophagy and suppressed the cells' viability ^35^. The treatment of human breast cancer cell lines with a cyclic tetrameric derivative of I3C resulted in upregulation of key signaling molecules involved in endoplasmic reticulum stress response and autophagy ^36^. I3C also has the potential to modulate the metabolism of estrogen, and, through this, it may lower the risk of hormone-dependent cancers ^37– 39^. In addition, I3C inhibited tumor invasion and metastasis ^40– 44^ and modulated the activity of several transcription factors and various protein kinases ^21^. Interestingly, I3C may also be involved in the inhibition of amyloid fibril formation ^45^. I3C was proposed to act as an angiogenesis agent, as it was shown to inhibit the development of new blood vessels ^46^. A number of studies have shown that I3C treatment leads to various changes in gene expression, including changes in key microRNAs (reviewed in 47). The complex mixture of indole metabolites found in cruciferous vegetables likely has synergistic anticarcinogenic effects that are not seen in experiments with individual compounds ^32^.

*In vivo* studies showed that I3C inhibits the development of different cancers in several animals when given before or in parallel to a carcinogen. However, when I3C was given to the animals *after* the carcinogen, I3C promoted carcinogenesis ^48^. This concern regarding the long-term effects of I3C treatment on cancer risk in humans resulted in some caution in the use of I3C as a dietary supplement in cancer management protocols ^49, 50^.

Mammalian cellular processes attributed to I3C action are as diverse as the different phenotypes presented by the many cancers studied. Indeed, focusing on the effects of I3C on one type of cancer (e.g. breast cancer) may present pleiotropic effects for I3C on multiple molecular targets (reviewed in 51) that may be distinct from those presented in another cancer type. While not often considered, to get a different perspective on the action of I3C in cells in general, it may be instructive to learn from the activity of I3C in plants.

## Indole-3-carbinol and plants

The model plant *Arabidopsis thaliana* provides an excellent system for elucidating the molecular mechanisms involved in I3C action, as 1) it produces I3C endogenously following herbivory, 2) small amounts of I3C are produced constitutively in the roots, hinting at an endogenous role in maintaining homeostasis, and 3) its short life cycle and small stature coupled with advanced available genetic and genomic resources make Arabidopsis an excellent model system not only for plant biology but also for eukaryotic research in general ^52^.

While the role of I3C in deterring herbivores is well studied ^53^, as is the biochemical pathway leading to the production of I3C ^13^, the secondary responses in plants induced by I3C are only now starting to be revealed. Our recent studies highlight that I3C is not only a defensive chemical targeting herbivores but also a signaling molecule modulating different cellular and developmental pathways.

Using Arabidopsis as a model system, we showed that exogenously applied I3C rapidly and reversibly inhibited root elongation in a dose-dependent manner ^54^. This inhibition was accompanied by three I3C-induced responses that are relevant for our understanding of I3C activity in inhibiting cancer.

**Figure 2.**
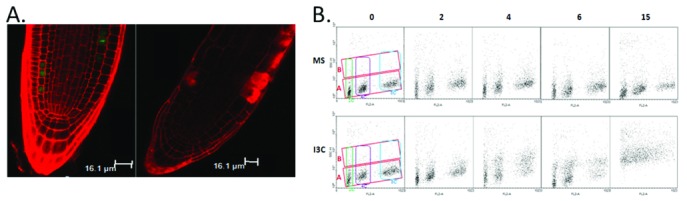
Indole-3-carbionol (I3C) affects cell cycle and nuclear complexity in the Arabidopsis root meristem. **A**. Confocal imaging reveals a lack of Cyclin B–GFP-expressing cells following I3C treatment. Seedlings expressing Cyclin B–GFP were grown on Murashige and Skoog (MS) medium for 4 days, treated with MS (left panel) or 500 μm I3C (right panel) for 6 hours, and imaged using confocal microscopy. Cell walls were stained using propidium iodide. **B**. Fluorescence-activated cell sorting (FACS) analysis reveals changes in nuclear complexity following I3C treatment. Nuclei were isolated from Arabidopsis roots treated with MS or MS plus I3C for the marked times between 0 and 15 hours and analyzed by FACS for DNA content (FL2-A = propidium iodide fluorescence) and nuclear complexity (SCC-H = light side scatter). The green, purple, and blue boxes represent the populations differing according to nuclear content, 2n, 4n, and the endoreplication population (8n), respectively. The red boxes represent two populations of nuclei (" **A**" and " **B**") that differ according to side scatter.

First, the application of I3C led to a cessation of cell division in the root meristem ( Figure 2A). While normally a number of CycB1-expressing cells are visible in the root meristem, following I3C treatment, no CycB1-containing cells were detected, indicating a cessation of cell division. This conclusion is supported by transcript-profiling results showing the downregulation of cell cycle genes six hours following exposure to I3C ^55^.

Fluorescence-activated cell sorting (FACS) analysis on nuclei isolated from root tips further indicates a stoppage of the cell cycle. As seen in Figure 2B, three distinct populations of nuclei are detected in untreated roots, corresponding to 2n, 4n, and 8n nuclei. However, following treatment with I3C, there is a progressive loss of the 4n and 8n populations, with a concurrent increase in cells with increased side-scatter (population B).

Second, the application of I3C led to a loss of auxin (indole-3-acetic acid [IAA]) activity in the root meristem ^54^. Auxin is the most central plant hormone, controlling nearly all aspects of plant growth and development ^56^. I3C affects plant growth and development by directly modulating auxin signaling. I3C antagonized a number of auxin-induced growth phenotypes, including inhibition of root elongation, formation of root hairs, and secondary root branching. I3C directly interferes with the auxin-dependent binding of the auxin-receptor Transport Inhibitor Response (TIR1) to two of its substrates ^54^. The TIR1 auxin receptor is an F-box-containing subunit of the SCF (Skp, Cullin, F-box) E3 ubiquitin ligase complex. Auxin binding to the SCF ^TIR1/AFB^ promotes the degradation of auxin/indole-3-acetic acid (Aux/IAA) transcriptional repressors and through this regulates the transcription of the auxin-induced genes ^57^. I3C inhibits the auxin-dependent dimerization of the receptor with its substrates by competing with auxin for the same binding site in TIR1.

The third I3C-induced response relevant for our understanding of I3C activity in inhibiting cancer is autophagy. Exposure of Arabidopsis roots to I3C leads to the induction of autophagy ^58^. This autophagy is not general, aimed at bulk degradation of general cytoplasmic content for recycling, such as that occuring under starvation conditions, but rather specific. Specific autophagy targets damaged proteins and other cellular components for degradation ^59^ and is seen in the co-localization of the GFP-AtATG8A and mCherry-AtNBR1 marker proteins in autophagosomes following I3C treatment. The I3C-induced autophagy targets the TIR1 auxin receptor, thus connecting I3C-dependent inhibition of auxin signaling ^55^ and the I3C induction of autophagy ^58^.

These two I3C-dependent processes were detected in roots not only after direct exposure to exogenously applied I3C but also following the treatment of leaves with I3C. Most importantly, leaf-wounding also induced autophagy and inhibited the auxin response in the root, and this effect of wounding was lost in glucosinolate-defective mutants. This indicates that an I3C-dependent signal is transported from leaves to the root meristem where auxin signaling is inhibited and autophagy is induced. Thus, I3C is not only a defensive metabolite that repels insects but also involved in long-distance communication regulating growth and development in plants.

## Indole-3-carbinol, autophagy, and protein turnover

The connection between I3C and autophagy is quite interesting, as this connection was also found in several human cancer cells, as described earlier ^35, 36^. The process of autophagy involves the degradation of unnecessary or dysfunctional cellular components through the actions of lysosomes (in mammals) or vacuoles (in plants). This process is evolutionarily conserved among eukaryotes, and its mechanism is well elucidated ^60, 61^. In the context of cancer, autophagy can be viewed as a “double-edged sword”. The activation of autophagy may function as a tumor suppressor (by degrading the defective organelles and cellular component) or can be exploited by the cancer cells to generate nutrients and energy during periods of starvation ^62^.

Although I3C-induced autophagy was detected in both plants and animals, the direct signaling mechanism has yet to be elucidated. However, this might hint at a shared signaling mechanism for both plants and humans. Thus, not only is it instructive for cancer biologists to learn from the activity of I3C in plants but also plant biologists have much to gain from a closer understanding of the mechanistic studies of cancer biologists.

To date, only a few I3C-binding proteins have been identified. In human cells, the enzyme elastase, which mediates the conversion of cyclin E from a higher- to a lower-molecular-weight form associated with cancer cell proliferation, was the first identified specific target protein for I3C ^63^. I3C treatments also inhibited elastase-dependent cleavage of an additional substrate, membrane-associated CD40, a member of the tumor necrosis factor (TNF) receptor superfamily ^64^. Thus, the I3C–elastase nexus may aid in the development of targeted therapies of human breast cancers where high elastase levels are correlated with poor prognosis.

The only I3C-binding protein identified in plants to date is the TIR1 F-box protein. While auxin is a plant-specific hormone, SCF complexes also exist in mammals and play important roles in many mammalian functions ^65^. TIR1 is related to the human protein SKP2 ^66^, so conceivably I3C could regulate protein turnover in mammals as well. This conjecture is supported by studies which showed that I3C targets and inhibits a different E3 ubiquitin ligase, NEDD4-1 (Neural precursor cell Expressed Developmentally Down regulated gene 4-1) in human melanoma cells ^67, 68^. Thus, an I3C-bound, inhibited NEDD4 would need to be cleared from the cell, and conceivably this could occur via specific autophagy, just as I3C-bound, inhibited TIR1 is targeted in plant roots for clearing by specific autophagy. As NEDD4 is frequently overexpressed in different types of human cancers ^69^, I3C could be a potential therapeutic agent inhibiting the activity of the over-accumulated E3 ligase.

While plants do not develop metastatic cancer as mammals do, plants can develop tumors. Plants and animals share numerous pathways and signaling cascades ^70^, and approximately 70% of the genes implicated in cancer have homologs in the *Arabidopsis thaliana* genome, similar to the percentages of human cancer genes in other established systems such as *Drosophila melanogaster*, *Caenorhabditis elegans*, and *Saccharomyces cerevisiae* ^52^. Furthermore, while plants and animals obviously have independent hormonal regulatory mechanisms, some similarity and cross reactivity exists between the kingdoms. Plants produce phyto-estrogens and other steroid hormones, which also affect human hormone signaling, as well as a number of putative steroid hormone-binding proteins ^71– 75^. Thus, the study of I3C in plants can have direct implications for further understanding the role of I3C and perhaps controlling cancer in humans.
